# Current Epidemiological Trends of Pediatric Traffic Accidents at a Romanian Clinical Hospital

**DOI:** 10.3390/children10091525

**Published:** 2023-09-08

**Authors:** Ștefan Popa, Ioan Sârbu, Carmen Iulia Ciongradi, Irene Paula Popa, Diana Bulgaru-Iliescu

**Affiliations:** 1 2nd Department of Surgery–Pediatric Surgery and Orthopedics, “Grigore T. Popa” University of Medicine and Pharmacy Iași, 700115 Iași, Romania; stefan.popa@umfiasi.ro (Ș.P.); carmen.ciongradi@umfiasi.ro (C.I.C.); 2 Surgery and Orthopaedic Clinic, “Sfânta Maria” Emergency Children Hospital Iași, 700309 Iași, Romania; 3 Department of Physiology, “Grigore T. Popa” University of Medicine and Pharmacy, 700115 Iași, Romania; 4 Cardiology Clinic, “St. Spiridon” County Clinical Emergency Hospital, 700111 Iași, Romania; 5 3rd Department of Medical Specialities–Legal Medicine, “Grigore T. Popa” University of Medicine and Pharmacy Iași, 700115 Iași, Romania; bulgarudiana@yahoo.com

**Keywords:** road traffic accidents, children, adolescents, orthopedic approach, fractures, medico-legal approach, safety

## Abstract

Background: Pediatric road traffic accidents (RTAs) have a substantial impact on the worldwide youth population, resulting in a considerable burden of disability. According to the World Health Organization’s (WHO) Global Status Report on Road Safety, around 1.35 million children die each year in RTAs around the world, having a big effect on health and financial costs. Today’s high-income countries like the Netherlands have experienced a decrease in the incidence of fatal traffic accidents (TAs) in children compared to countries with higher-than-average scores, including Romania, where roughly one out of every two minor deaths was a pedestrian; however, there is a lack of comprehensive and up-to-date epidemiological data on non-fatal TAs regarding pediatric patients. The objective of this study is to perform a thorough examination of the epidemiological aspects of Tas in pediatric patients admitted to the Emergency Department (ED) of “St. Mary’s” Emergency Clinical Hospital for Children in Iasi, Romania. Materials and methods: A descriptive retrospective research study was conducted at the “St. Mary’s” Emergency Clinical Hospital for Children in Iasi, Romania, from January 2015 to December 2022. The research population includes all pediatric trauma patients that were between the age range of 1 month and 18 years who were treated by the trauma department. A total of 358 cases met the inclusion criteria and fulfilled fulfilled fulfilled. Data concerning variables such as accident incidents, types of injuries, and length of hospitalization have been gathered. Results: The average age of the patients was 11.43 ± 4.07 years, with patients of both sexes, the representation of the male sex being 78.5%. The incidence occurred during the summer, representing 15.3% in June. Of the patients admitted to the ED, 55.5% (n = 196) did not require surgery. Most of the patients spent from a minimum of one day to a maximum of 28 days in the hospital, with an average of 8.50 hospital days. The most common injuries were fractures (n = 221), and the most frequent anatomical region affected was the upper limbs (n = 55.2%). Conclusion: While the literature on fatal TA cases shows a declining trend, there is a lack of up-to-date information on non-fatal TAs involving children. The results of our study suggest that there is a high incidence of pediatric TAs due to the scale of “St. Mary’s” Emergency Clinical Hospital for Children, from Iasi, which provides medical services to a considerable number of patients coming from both rural and urban areas of the seven counties of Moldova region, in Romania.

## 1. Introduction

Pediatric traffic accidents (TAs) are a matter of significant public health concern on a worldwide basis, leading to a substantial number of fatalities and disability among children and adolescents [1]. According to the World Health Organization (WHO), road traffic accidents (RTAs) are identified as the main cause of death among those aged 14–18 and the second most prevalent cause among those aged 4–13 [2].

The projected worldwide fatality count resulting from RTAs is around 1.35 million, as reported in sources [3,4]. In accordance with the European Road Safety Observatory [5], the overall total of road casualties among minors dropped by 47% between 2011 and 2020, the most significant reduction in mortality being recorded among those aged 5 to 9 years in high-income countries, because of increased parental supervision, safety education programs for children and improved road safety measures. This study, which analyzed statistics from the past decade, highlights the concerning disparity in child road safety between Romania and other European nations [6,7]. Despite a decline in morbidity and mortality associated with TA in wealthy nations, Romania has a rate of 27 cases per 1 million inhabitants under the age of 18, which is seven times higher compared to countries like Great Britain, Norway, and Sweden, thus remaining a significant contributor to disability-adjusted life years (DALYs) among individuals aged 12–18, making up more than 7% of DALYs. Among younger children, TAs are responsible for almost 3% of DALYs and serve as the primary source of injury-related fatalities [8,9,10,11]. According to another study conducted by the European Transport Safety Council between 2018 and 2020, Romania ranks highest in Europe in terms of the number of road accident-related deaths among children under the age of 14, with an average rate of 19.1 cases per 1 million. However, Romania is not alone, being followed by other countries with higher-than-average scores, like Bulgaria, with an average rate of 17.3 cases per 1 million, and Latvia, with an average rate of 11.1 cases per 1 million.

In addition to the profound physical and cognitive impairments, individuals with substantial disabilities impose significant economic burdens [2,6,12]. A study from 2018 stated that for around 38,000 children injured or killed in vehicle TAs in the United States, USD 55 billion are spent each year on medical care costs, in addition to the immeasurable burden on the victim’s family and friends [13]. While there is existing knowledge regarding the association between TAs and fatal consequences, there is a lack of subsequent research examining the non-fatal injuries resulting from TAs in mid-income countries because the majority of epidemiological studies on TAs have primarily concentrated on adults and fatal injuries, with an emphasis on both low-income and wealthy economies. Addressing this gap and devising practical strategies for enhancing injury prevention is of the utmost importance [13,14].

A significant proportion of vehicle-related deaths can be ascribed to vulnerable road users, including pedestrians and cyclists. According to the cited sources [15,16,17], the percentage of pedestrian casualties was higher than 64.9% compared to vehicle occupants, which accounted for around 14.9% of the total. According to other studies, a significant proportion of pedestrians who sustain severe injuries in motor vehicle incidents were individuals in the pediatric age group, often ranging from 16 to 20 years old. Since 1979, there has been a global initiative aimed at mitigating fatal road accidents across all demographic groups [18,19]. Based on an annual evaluation conducted by the Organization for Economic Cooperation and Development, there was a 15% reduction in the overall number of road deaths from 2010 to 2014. This fall is similar to the decrease observed during the period from 2006 to 2010. However, this ongoing trend continues to exhibit variability. According to a survey in January 2016, there was a 2.4% rise in road deaths in 2015, marking the second consecutive year of growth. In contrast, there was a 3.5% decline in the percentage of trauma events [19].

Musculoskeletal lesions are infrequently associated with possible fatality, yet they have the capacity to induce significant sequelae [20,21]. Pediatric orthopedic surgeons are often extensively involved in their practice due to the fact that 76% of children with multiple injuries suffer from extreme trauma [22]. Furthermore, the presence of bone trauma, including injuries to the spine, clavicle/scapula, femur, and pelvis, necessitates the provision of critical care and prolongs the duration of hospitalization [23,24].

The goal of this study was to conduct a detailed analysis of children (between the ages of 18 years and under) who received medical treatment in an emergency department (ED) as a consequence of TAs in a country with middle-income levels. The main purpose was to ascertain future research regions that may be the subject of future preventative interventions to mitigate TAs involving children. According to prior research [14,25,26], there was a disproportionate representation of male participants in the selected population.

## 2. Materials and Methods

The present study constitutes a retrospective analysis that was carried out over a span of eight years, specifically from 2015 to 2022. The main aim of this study was to examine the numerous risk variables that are linked to injuries sustained in TAs by children and adolescents. The study utilizes patient medical records obtained from the Emergency Department of “St. Mary’s” Emergency Clinical Hospital for Children in Iasi, Romania, as well as hospitalization data from the Pediatric Orthopedics Department of the same hospital.

The study protocol received permission from the administration of “St. Mary’s” Clinical Emergency Hospital for Children in Iasi, with the assigned approval number 23177/10.07.2020.

The Emergency Department (ED) of the St. Mary’s Clinical Emergency Hospital for Children in Iasi receives around 35,000 visits every year. Among these visits, approximately 4% are related to trauma cases, with 54% of those specifically involving children between the ages of 7 and 14 years. Between 24 and 71 road traffic accidents, with an average of 44.75 cases per year, are treated in our hospital. The ED serves as the primary public referral center for pediatric injuries, providing highly specialized medical care for the municipality and county of Iasi as well as for the 7 counties of Moldova, including rural as well as urban areas.

The inclusion criteria were patients between the ages of 1 month and 18 years involved in RTAs investigated in the ED) The information from the hospital’s computerized medical records was extracted and specifically focused on those assigned cause codes V00–V99. In addition, we performed an electronic search of the medical records with the terms “road traffic accidents” and “traffic accidents” to identify any instances of TAs that may have been overlooked in the categorization process based on given cause codes.

Following the initial screening process, the patients who were included in the research underwent a meticulous manual verification process to confirm their status as TA patients. A TA was defined in the study as an accident occurring on a road involving at least one moving vehicle. The research eliminated injuries that were not related to a moving vehicle. A total of 358 cases were revealed. The process of data collection encompassed the acquisition of data on demographics, comprehensive information about the kinds of injuries incurred, and the treatments administered. The classification of various types of accidents was determined by the child’s location at the time of the incident, whereas the classification of injury types was based on the specific anatomical region injured and the nature of the damage. In order to conduct the statistical evaluations, the subjects were categorized into separate age groups, such as (a) preschool age (0–6 years), (b) pre-adolescence (7–14 years), and (c) adolescence (15–16 years).

The statistical analysis of the study data was conducted using SPSS Statistics, Version 26.0. The continuous variables were represented using statistical measures such as the mean, median, mode (representing the dominant value), standard deviation, skewness coefficient, kurtosis coefficient, minimum, and maximum. The categorical variables were represented using absolute frequencies and percentages. The investigation of the relationship between categorical variables was performed by employing cross-tabulation tables and doing the χ2 (chi-square) test. When the outcomes of the chi-square test were substantially modified and deemed unreliable, Fisher’s exact test was employed as an alternative. The One-Way ANOVA approach was utilized to compare the means of parameters across different groups. Subsequently, the Bonferroni posthoc test was performed to conduct multiple comparisons. A statistical significance level of *p* < 0.05 was deemed to be significant.

## 3. Results

A total of 358 cases met the inclusion criteria for the present research.

### 3.1. Age and Gender Distribution

**Figure 1:**
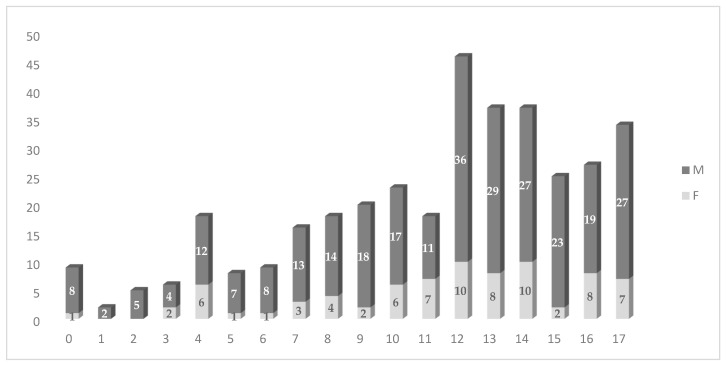
Histogram of the age and gender distribution of the study population for 0–17 year-old traffic accident patients attending the Emergency Department (ED) of “St. Mary’s” Emergency Clinical Hospital for Children, Iasi, Romania (January 2015 to December 2022).

A substantial difference in both gender and age distribution was seen regarding the types of accidents. The study group consists of individuals from both genders, with males accounting for 78.5% of the population and females for 21.5%. The mean age of the participants in the sample is 11.43 years, with a standard deviation of around ±4.07 years. The average, or the most often observed age in the group in question, is 12 years. The ages documented in the dataset span from a minimum of one month to a maximum of seventeen years. The age distribution has a negative skewness, as evidenced by a coefficient of −0.634, showing a rightward inclination. Furthermore, there are several data points that deviate significantly toward the lower end of the distribution. In addition, the distribution may be classified as platykurtic based on its kurtosis coefficient of −0.357 (Figure 1).

### 3.2. Annual Patient Report

The current investigation, conducted over a duration of eight years, especially from 2015 to 2022, reveals a peak in cases in 2018, accounting for 20.1% of the total cases, and the lowest recorded number in 2017, amounting to 6.8%. The data shown in Table 1 illustrate that the percentage fluctuates on an annual basis, with a notable downward trend observed in the most recent three-year period.

### 3.3. Types of Accidents

Approximately 30.8% of the recorded accidents featured a cyclist, while 24.3% of the incidents involved pedestrians. Another 24% involved passengers of a different nature, but 12.1% of the incidents were attributed to someone falling from a horse. Lastly, 8.8% of the accidents involved a passenger within a motor vehicle. The objective of our study was to ascertain if certain categories of accidents necessitated the need for surgical intervention. It was discovered that irrespective of the kind of injury, a comparable percentage ranging from 39.5% to 49.4%) required surgical intervention, as evidenced by the data presented in the table. To evaluate possible associations between the kind of accidents and several indicators, such as the cumulative duration of hospitalization and the overall quantity of X-rays, we utilized the One-Way ANOVA technique. The findings of the study revealed that the mean duration of hospitalization varied between 6.90 and 9.84 days, with no statistically significant variation seen among the various categories of accidents. Regarding the aggregate quantity of X-rays, it was discovered that the mean value varied between 6.16 and 7.50, regardless of the nature of the incident (Table 2).

### 3.4. Seasonal Variation

The distribution of TAs exhibited distinct variations according to the seasons; the summer, starting with June (15.3%) and July (10.2%), being the months characterized by the highest number of presentations in the ED, closely followed by the months after vacation, April (11.9%) and October (11%). In contrast, it is seen that the winter months of December (4.8%), February (3.1%), and January (1.7%) had the lowest incidence of instances. Moreover, TAs were mostly present during times when children started their schooling, were on vacation, or participated in recreational activities.

### 3.5. Duration of the First Hospitalization

**Figure 2:**
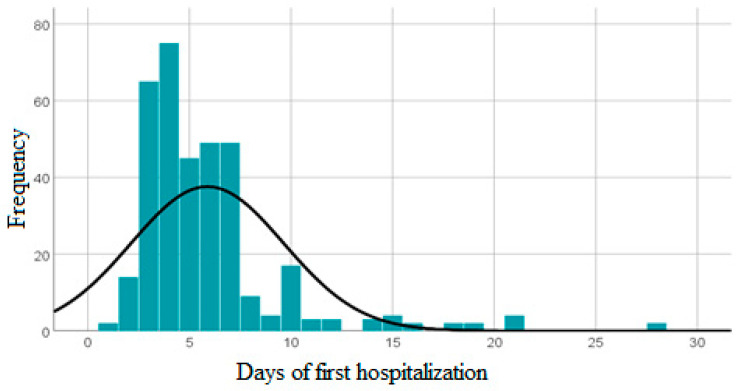
Represents a histogram that illustrates the distribution of instances based on the time of year for the patients included in the research at “St. Mary’s” Emergency Clinical Hospital for Children, Iasi, Romania (January 2015 to December 2022).

Upon analysis of the sample, it was determined that the average duration of the first hospitalization was 5,88 days. The observed range of values for the variable under consideration spanned from a minimum of one day to a maximum of 28 days. The duration of the initial hospitalization varies from the mean value by a standard deviation of 3.75 days (Figure 2).

The value of the bolt is 9592, which suggests that the distribution is leptokurtic. The coefficient of asymmetry is calculated to be 2666, indicating a positively skewed, left-leaning asymmetric distribution with a greater concentration of extreme values toward the right side.

### 3.6. Treatment

Approximately 44.5% of the individuals affected by vehicular collisions received surgical interventions. Among these patients, 55% required a single surgical treatment, 33.3% necessitated two surgeries, 11.1% required three surgeries, and a small proportion of 0.6% even required four surgeries. Among the surgical cases under consideration, it was found that 59.8% were conducted as emergency operations, while 38.5% were performed within a time frame of two days. A very small proportion of 1.7% happened after a period of two weeks. As stated earlier, a majority of the patients, namely 55.5%, did not necessitate surgical intervention. However, within this subgroup, a significant proportion, precisely 61.6%, still required orthopedic reduction and immobilization with the use of sedation (as indicated in Table 3).

## 4. Discussion

Our study benefits from a lengthy 8-year data collection period (from 2015 to 2022) and a large sample size (358 cases of childhood TAs examined), of which a significant proportion, namely 55.5% of the patients, did not require surgical intervention. Within this particular group, a sizable portion of people (61.6%) required orthopedic intervention involving reduction and immobilization along with anesthesia. It is noteworthy that the majority of these injuries were observed in the upper extremities. It is also important to point out that a significant proportion of accidents, specifically 30.8%, were recorded in children who were under the age of 14. However, an obvious shift in the frequency and characteristics of accidents was seen subsequent to reaching the age of 15. Within the demographic of individuals aged 15 to 17, falls from horses emerged as the most predominant form of accident. The incidence of most TAs was seen to be concentrated in the summer months, maybe because of the advantageous weather conditions that facilitate pedestrian and cycling activities. It is important to highlight that previous academic investigations have mostly concentrated on the lethal part of TAs or just automobile collisions, therefore distinguishing our study’s design in terms of its coverage and methodology.

The ED of St. Mary’s Emergency Clinical Hospital for Children, Iasi, Romania, provides medical services to a considerable number of patients coming from both rural and urban areas of the seven counties of Moldova. As a result of this study, the findings possess generalizability to other counties of Romania.

All patients brought to the Emergency Department (ED) with traffic-related injuries were included. In contrast to other studies that frequently limited their focus to children aged 12 and under or from 14 to 20 years old, this study used more comprehensive inclusion criteria to accommodate the increased incidence of reported incidents. Several discoveries from our investigation correspond with those of previous studies.

Gender distribution seen in our study (with males accounting for 78.5% of the population and females for 21.5%) aligns with findings from previous research [4,7], indicating a significant bias towards men.

The observed distribution of injuries resulting from the rise in road traffic accidents, as shown in our study, differs from prior research findings. This discrepancy might perhaps be attributed to the inclusion of patients treated with orthopedic reduction and immobilization, as well as surgical interventions. It is worth noting that the majority of bicycle-related injuries documented in our study were of a moderate to severe type. In contrast, a reduced incidence of accidents was noted among individuals traveling by car, potentially attributable to alterations in legal regulations and developments in safety technology within the automobile industry [4,8,13].

Seasonal fluctuations were also documented but to a lesser extent than in our study. The observed discrepancy may be plausibly ascribed to Romania’s geographical position in the eastern region of Europe, having a temperate–continental climate of a transitional type, specific to Central Europe. The prevalence of horse riding (in our country, the horse being used more as a means of traction for unmotorized vehicles) and bicycle usage experiences a notable reduction during the winter season [5,11].

To assess the presence of significant associations between the kind of accident and several indicators, including the total duration of hospitalization, and recovery time, we employed the ANOVA One-Way variance analysis.

The findings of the study indicate that there exists a statistically significant discrepancy among the various types of accidents alone about the duration required for recovery (*p* = 0.024). Regarding the aggregate duration of hospital stays, the mean falls within the range of 6.90 to 9.84 days, with no statistically significant variations seen among different accident categories. The Bonferroni test assesses the significant differences among the five types of accidents. The findings revealed notable disparities in the duration of recovery among pedestrians and cyclists, with average recovery periods of 7.14 months and 8.39 months, respectively. In contrast, individuals who experienced a horse fall exhibited an average recovery time of 5.23 months.

A statistically significant correlation has been shown between the nature of the injury and the necessity of surgical intervention. In instances of ligament ruptures, concussions, and locations, the necessity for surgical intervention is notably minimal. However, in the case of fractures, the likelihood of requiring surgery increases significantly. Specifically, no less than 39.8% of individuals with fractures necessitate surgical procedures. Moreover, in situations involving wounds or fractures accompanied by wounds, the demand for surgical intervention is notably elevated, with rates reaching 79.2% and 77.1%, respectively.

Additionally, we have analyzed the correlation between the kind of injury and indicators like the overall count of radiological exams, total duration of hospitalization, and the time required for recovery. The findings indicate that there are statistically significant disparities across the various injury types regarding the three aforementioned variables.

Regarding the overall quantity of X-rays, it is evident that individuals presenting with both fractures and wounds required the highest average number of X-rays, standing at 10.29. Subsequently, those with fractures alone and those with wounds alone followed in terms of X-ray requirements.

Regarding the aggregate duration of hospitalization, individuals concurrently afflicted with wounds and fractures exhibited a notably elevated mean number of hospital days in comparison to patients with alternative forms of injuries.

Regarding the duration of healing, those suffering from fractures and infections, as well as those with ligament ruptures, faced the longest recuperation times. Individuals with wounds, concussions, and locations exhibited the shortest healing time.

The relationship between the affected limb, the overall length of hospitalization, and the amount of time needed for recovery was also investigated. The findings indicate that there exists a statistically significant disparity, mainly in the duration of hospitalization days among the afflicted limbs (*p* < 0.001). Individuals with impaired lower limbs required an average hospitalization period of 10.32 days, whereas those with impaired upper limbs necessitated an average hospital stay of 7.11 days.

There is no statistically significant disparity observed in the total count of X-rays and the duration of recovery between individuals with upper limb injuries and those with lower limb injuries.

The results of this study emphasize the significance of establishing safety measures aimed at safeguarding children in their roles as pedestrians, bikers on roadways, or passengers. However, further studies are necessary to determine the most effective preventive measures.

## 5. Conclusions

The findings of our study indicate a substantial prevalence of pediatric TAs during the pre-adolescent and adolescent ages. Due to Romania’s geographical position in the eastern region of Europe and the scale of “St. Mary’s” Emergency Clinical Hospital for Children in Iasi, the hospital provides medical services to a considerable number of patients coming from both rural and urban areas of the seven counties of the Moldova region in Romania.

It is noteworthy that a considerable percentage of persons (61.6%) required only orthopedic intervention, namely reduction and immobilization, which are often performed under anesthesia. Based on our comprehension, the key causes that contribute to these injuries are mostly observed during the age range of 7–14, with the individuals affected mainly being bikers.

It is our desire that future initiatives should focus on increasing the safety of both pedestrians and bicyclists by enforcing traffic laws, implementing traffic control measurements, improving road infrastructure, increasing visibility with better street illumination, encouraging driver education programs, and installing cutting-edge driver assistance systems. Promoting and integrating the use of child restraint devices for passengers in vehicles is one of the other directions.

## Figures and Tables

**Table 1:** Annual distribution of patients between the ages of 1 month and 17 years involved in TAs that attended the Emergency Department (ED) of “St. Mary’s” Emergency Clinical Hospital for Children, Iasi, Romania (January 2015 to December 2022).

Year of Presentation	Frequency	Percent
2015	39	11.0
2016	57	16.1
2017	24	6.8
2018	71	20.1
2019	37	10.5
2020	61	17.2
2021	38	10.7
2022	31	7.6
Total	358	100.0

**Table 2:** The distribution of radiological tests conducted during hospitalization for children in different age groups (0–17 years) who were involved in TAs and sought medical attention at the Emergency Department (ED) of “St. Mary’s” Emergency Clinical Hospital for Children in Iasi, Romania (data collected spans from January 2015 to December 2022).

AGE GROUPS								
	0–6 Y.O.		7–14 Y.O.		15–18 Y.O.		TOTAL	
	N	%	N	%	N	%	N	%
GENDER								
Male	46	12.85	165	46.08	69	19.28	280	78.21
Female	11	3.08	50	13.96	17	4.75	78	21.79
TOTAL	57	15.93	215	60.04	86	24.03	358	100
ACCIDENT TYPE								
Pedestrian	17	4.74	52	14.52	18	5.04	87	24.30
Bicycle	13	3.63	86	24.02	10	2.80	109	30.44
Car passenger	0	0	29	8.10	2	0.56	31	8.66
Fall off a horse	13	3.63	5	1.39	26	7.28	44	12.30
Other passengers	14	3,92	55	15,36	18	5.02	87	24.30
TOTAL	57	15.92	227	63.40	74	20.68	358	100
INJURIES								
ANATOMICAL REGION								
Upper limb	26	7.28	131	33.60	42	14.92	199	55.58
Lower limb	31	8.65	81	22.62	44	12.32	156	43.59
Head	0	0	2	0.55	1	0.28	3	0.83
TOTAL	57	15.93	214	59.77	87	24.30	358	100
INJURY TYPE								
Fracture	45	12.57	170	47.48	66	18.45	281	78.5
Contusion	4	1.12	14	3.91	6	1.67	24	6.7
Wound	7	1.95	27	7.54	13	3.63	47	13.12
Ligament rupture	0	0	2	0.56	1	0.28	3	0.84
Sprain	1	0.28	2	0.56	0	0	3	0.84
TOTAL	57	15.92	215	60.05	86	24.03	358	100

Y.O. = Years Old.

**Table 3:** In ages taken during hospitalization and operations number of patient groups for 0–17-year-old traffic accident patients attending the Emergency Department (ED) of “St. Mary’s” Emergency Clinical Hospital for Children, Iasi, Romania (January 2015 to December 2022).

AGE GROUPS								
	0–6 Y.O.		7–14 Y.O.		15–18 Y.O.		TOTAL	
	N	%	N	%	N	%	N	%
X-ray								
1	23	1	57	2.46	33	1.42	113	4.88
2 or more	410	17.72	1288	55.63	504	21.77	2202	95.12
TOTAL X-ray	433	18.70	1345	58.1	537	23.20	2315	100
CT								
1	16	31.45	12	23.54	17	33.33	43	84.32
2 or more	3	5.80	1	1.96	2	3.92	8	15.68
TOTAL CT	19	37.25	13	25.50	19	37.25	51	100
MRI								
1	1	25	2	50	1	25	3	100
2 or more	0	0	0	0	0	0	0	0
TOTAL MRI	1	25	2	50	1	25	4	100
TOTAL	453	19.11	1360	57.39	557	23.50	2370	100
OPERATIONS DURING HOSPITALIZATION								
None	34	9.51	124	34.64	42	11.75	200	55.5
1	6	1.67	19	5.30	9	2.51	34	9.87
2 or more	17	4.74	72	20.11	35	9.77	124	34.63
TOTAL OPERATIONS COUNT	57	15.92	215	60.05	86	24.03	358	100

## Data Availability

The data presented in this study are available on reasonable request.
